# Protein Kinase C Delta (PKCδ) Affects Proliferation of Insulin-Secreting Cells by Promoting Nuclear Extrusion of the Cell Cycle Inhibitor p21^Cip1/WAF1^

**DOI:** 10.1371/journal.pone.0028828

**Published:** 2011-12-27

**Authors:** Felicia Ranta, Johannes Leveringhaus, Dorothea Theilig, Gabriele Schulz-Raffelt, Anita M. Hennige, Dominic G. Hildebrand, René Handrick, Verena Jendrossek, Fatima Bosch, Klaus Schulze-Osthoff, Hans-Ulrich Häring, Susanne Ullrich

**Affiliations:** 1 Division of Endocrinology, Diabetology, Vascular Medicine, Nephrology and Clinical Chemistry, Department of Internal Medicine, University of Tübingen, Tübingen, Germany; 2 Interfaculty Institute for Biochemistry (IFIB), University of Tübingen, Tübingen, Germany; 3 Institute for Pharmaceutical Biotechnology, Biberach University of Applied Sciences, Biberach, Germany; 4 Institute of Cell Biology, University of Essen, Essen, Germany; 5 Center of Animal Biotechnology and Gene Therapy, Universita Autònoma Barcelona, Bellaterra and CIBER de Diabetes y Enfermedades Metabólicas Asociadas (CIBERDEM), Barcelona, Spain; University of Bremen, Germany

## Abstract

**Background:**

High fat diet-induced hyperglycemia and palmitate-stimulated apoptosis was prevented by specific inhibition of protein kinase C delta (PKCδ) in β-cells. To understand the role of PKCδ in more detail the impact of changes in PKCδ activity on proliferation and survival of insulin-secreting cells was analyzed under stress-free conditions.

**Methodology and Principal Findings:**

Using genetic and pharmacological approaches, the effect of reduced and increased PKCδ activity on proliferation, apoptosis and cell cycle regulation of insulin secreting cells was examined. Proteins were analyzed by Western blotting and by confocal laser scanning microscopy. Increased expression of wild type PKCδ (PKCδWT) significantly stimulated proliferation of INS-1E cells with concomitant reduced expression and cytosolic retraction of the cell cycle inhibitor p21^Cip1/WAF1^. This nuclear extrusion was mediated by PKCδ-dependent phosphorylation of p21^Cip1/WAF1^ at Ser146. In kinase dead PKCδ (PKCδKN) overexpressing cells and after inhibition of endogenous PKCδ activity by rottlerin or RNA interference phosphorylation of p21^Cip1/WAF1^ was reduced, which favored its nuclear accumulation and apoptotic cell death of INS-1E cells. Human and mouse islet cells express p21^Cip1/WAF1^ with strong nuclear accumulation, while in islet cells of PKCδWT transgenic mice the inhibitor resides cytosolic.

**Conclusions and Significance:**

These observations disclose PKCδ as negative regulator of p21^Cip1/WAF1^, which facilitates proliferation of insulin secreting cells under stress-free conditions and suggest that additional stress-induced changes push PKCδ into its known pro-apoptotic role.

## Introduction

Sufficient β-cell mass is required for adequate insulin secretion. Consequently, an elevated demand of insulin is controlled by increased proliferation of pancreatic endocrine cells while insufficient insulin secretion and the development of type-2 diabetes have been associated with β-cell death [1]. A variety of molecular changes are involved in β-cell failure including reduced insulin/IGF-1 receptor signaling, endoplasmic reticulum stress and mitochondrial dysfunction [2]–[10]. These changes are triggered by obesity-linked factors, such as oxidative stress, saturated free fatty acids, cytokines and interleukins. Previous observations from our and other groups suggested that protein kinase C delta (PKCδ) plays a decisive role in β-cell failure induced by cytokines and free fatty acids [11]–[15]. Thus, mice with targeted overexpression of a kinase-negative PKCδ (PKCδKN) mutant in β-cells are protected against high fat diet-induced glucose intolerance and show increased survival of islet β-cells [14]. Conversely, we have previously shown that exposure of β-cells to high concentrations of palmitate promotes PKCδ-mediated nuclear accumulation of FOXO1, a pro-apoptotic transcription factor activated under stress conditions [14]. Furthermore, PKCδ has been found to mediate iNOS mRNA stabilization induced by IL-1β, whereas ablation of PKCδ protected mice against streptozotozin-induced hyperglycemia [11], [12]. Thus, under certain stress conditions, PKCδ promotes signaling pathways leading to apoptotic β-cell death.

Very few studies have investigated the role of PKCδ for normal β-cell function, in particular under stress-free conditions. Surprisingly, mice with increased transgenic expression of PKCδ in β-cells develop and age normally under chow diet and maintain normal glucose tolerance (unpublished observations). As a matter of fact, although PKCδ can serve as a pro-apoptotic signal, depending on the cellular context, it can also elicit anti-apoptotic and survival signals in a variety of cell systems [16]–[18]. These proliferative effects might involve a direct interference of PKCδ with cell cycle regulation [19], [20]. Intriguingly, proliferation of differentiated β-cells is a rare event although proteins which are important for cell cycle progression are expressed [21]. In adult mice less than 0.4% of β-cells stain positive for BrdU, in cultured human islet preparations only 0.3% of the cells proliferate [21]–[23]. Proliferation is tightly controlled by the sequential expression and activation of cell cycle regulators, such as cyclins and cyclin-dependent kinases (CDKs). The mitogenic activity of cyclin-CDK complexes is limited through binding of transiently expressed cell cycle inhibitors [24]. Inhibitors of the Cip/Kip family, p21^Cip1/WAF1^, p27^kip1^ and p57^Kip2^ are ubiquitously expressed proteins that slow down proliferation and cell cycle progression at G1/S or G2/M phase transitions [25]. While p57^Kip2^ regulates cell cycling mainly during development, p21^Cip1/WAF1^ and p27^kip1^ accumulate in mitogen-starved cells and mediate cell cycle arrest upon DNA damage [26]–[28]. In accordance with a minor role of p21^Cip1/WAF1^ during development, mice deficient of p21^Cip1/WAF1^ show normal growth and differentiation of the endocrine pancreas [22]. In contrast, mice that specifically overexpress p21^Cip1/WAF1^ in β-cells have impaired β-cell replication and develop age-related hyperglycemia due to increased apoptosis [29].

The activity of p21^Cip1/WAF1^ is regulated further by its subcellular distribution which is controlled by phosphorylation of p21^Cip1/WAF1^ at residues located in the C-terminal domain in proximity to the nuclear localization sequence [30]. PKB/Akt-mediated phosphorylations at Ser146 and at Thr145 sequester p21^Cip1/WAF1^ into the cytosol [31]. *In vitro* phosphorylation assays have further shown that PKCδ can phosphorylate directly p21^Cip1/WAF1^ at Ser146, which triggers its cytosolic accumulation and influences the stabilization of p21^Cip1/WAF1^ [20].

In the present study, we examined the role PKCδ plays in proliferation and survival of insulin-secreting cells. Our results suggest that PKCδ phosphorylates the cell cycle inhibitor p21^Cip1/WAF1^ at Ser146, which favors its nuclear extrusion and supports proliferation under stress-free conditions. However, under stress conditions such as free fatty acids PKCδ turns into a pro-apoptotic kinase.

## Results

### PKCδ affects proliferation and apoptosis of insulin-secreting cells

**Figure 1:**
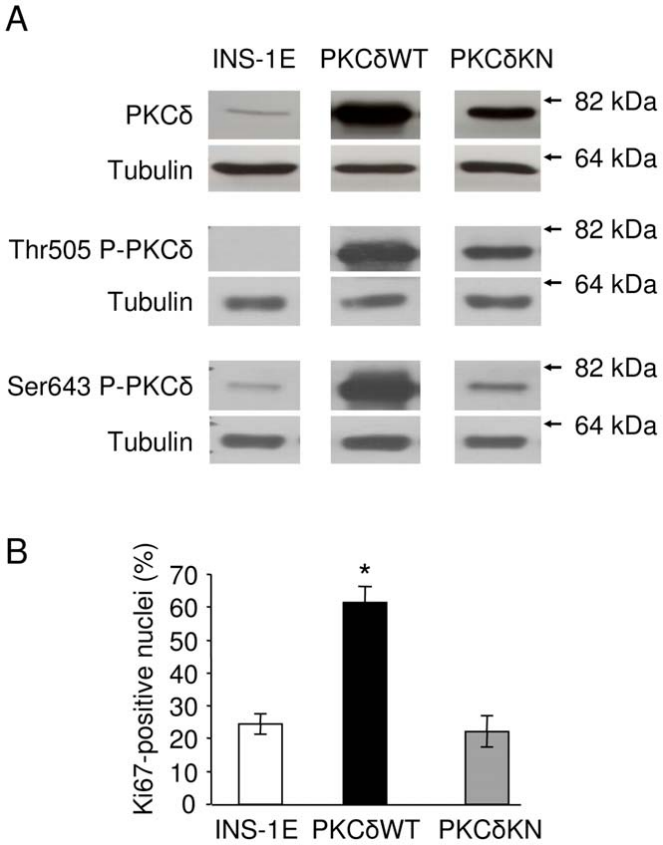
Under non-stress condition PKCδ promotes proliferation of insulin secreting INS-1E cells. (A) Representative Western blots demonstrating expression and phosphorylation at Thr505 and at Ser643 of PKCδ of control INS-1E cells and cells overexpressing PKCδWT or PKCδKN. Tubulin was used as loading control. Molecular weight markers are shown on the right. (B) Percentage of control, PKCδWT and PKCδKN INS-1E cells staining positive for Ki67 after 2 d culture. Data are expressed as means ± SEM of n = 3–4 independent experiments. * (p<0.05) represents significance to control cells.

The first observation that PKCδ may influence cell growth was made with INS-1E cells which were transfected with either an active PKCδ (PKCδWT) or an inactive, kinase dead PKCδ (PKCδKN, Fig. 1A). Surprisingly, PKCδWT transfected cells displayed 2.4 times more nuclei stained positive for the proliferation marker Ki67 when compared to untransfected control cells (Fig. 1B). The phosphorylation of PKCδ at Ser643 and Thr505 was increased proportionally to the protein amount in PKCδWT INS-1E cells under standard culture conditions, which is indicative for an active PKCδ (Fig. 1A). In PKCδKN INS-1E cells phosphorylation of PKCδ at Thr505, a phosphorylation site of PDK1, is also increased proportionally to the protein amount, while phosphorylation at the autophosphorylation site Ser643 is reduced (Fig. 1A) [32]–[34]. It is noteworthy that the PKCδKN mutant remains inactive regardless of the degree of phosphorylation. These observations suggest that PKCδ supports proliferation of INS-1E cells.

### PKCδ controls cytosolic-nuclear trafficking of p21^Cip1/WAF1^ in INS-1E cells and primary mouse islet cells

**Figure 2:**
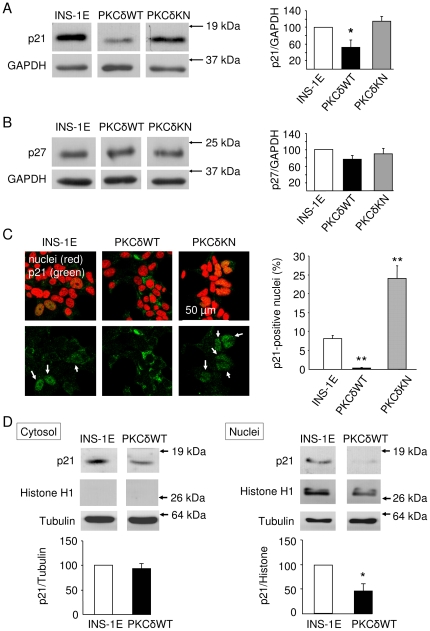
Nuclear-cytosolic distribution of the cell cycle inhibitor p21^Cip1/WAF1^ depends on PKCδ in INS-1E cells. Representative Western blots of (A) p21^Cip1/WAF1^ and (B) p27^kip1^ expression in control, PKCδWT and PKCδKN INS-1E cells and means + SEM of n = 3 independent experiments of the amounts of p21^Cip1/WAF1^ and p27^kip1^ relative to GAPDH as determined by densitometry. * (p<0.05) represents significance to control INS-1E cells. (C) Subcellular distribution of p21^Cip1/WAF1^ protein as analyzed by confocal laser scanning microscopy. Nuclei are stained in red, p21^Cip1/WAF1^ in green. The percentage of p21^Cip1/WAF1^-positive nuclei is expressed as means + SEM of n = 3–4 independent experiments. ** (p<0.01) represents significance against control INS-1E cells. (D) Representative Western blots and relative quantities expressed as means + SEM of n = 3 independent experiments of p21^Cip1/WAF1^ in cytosolic and nuclear fractions of control and PKCδWT INS-1E cells. * (p<0.05) indicates significance to p21^Cip1/WAF1^ of control INS-1E cells set to 100%.

The analysis of proteins which regulate proliferation revealed that PKCδWT cells expressed a significantly lower amount of the cell cycle inhibitor p21^Cip1/WAF1^ than control or PKCδKN INS-1E cells, while no change in expression of p27^kip1^ was apparent (Fig. 2A, B). Notably, due to the shorter length of rodent p21^Cip1/WAF1^ compared to the human orthologue, the protein band of p21^Cip1/WAF1^ displayed an apparent molecular weight lower than 21 kDa. This band was specific for p21^Cip1/WAF1^, as a protein of the same size was detected in bleomycin-treated wild type mouse embryonic fibroblasts (MEFs) but not in p21^Cip1/WAF1^-deficient MEFs (Fig. S1A). Moreover, the specificity of two antibodies used in this study was confirmed by immunocytochemistry, which revealed a p21^Cip1/WAF1^-specific staining in bleomycin-treated MEFs that was completely absent in untreated cells or in p21^Cip1/WAF1^-deficient cells exposed to the DNA damaging agent (Fig. S1B). The reduced expression of p21^Cip1/WAF1^ in PKCδWT INS-1E cells may be responsible for accelerated proliferation.

Even more interesting is that the analysis of the subcellular distribution of p21^Cip1/WAF1^ using confocal laser scanning microscopy showed that PKCδWT cells expressed p21^Cip1/WAF1^ almost exclusively in cytoplasm. In contrast, in PKCδKN cells and to a lesser extent in control INS-1E cells p21^Cip1/WAF1^ nuclear accumulation of the inhibitor was apparent (Fig. 2C). The reduced nuclear localization of p21^Cip1/WAF1^ in PKCδWT cells was confirmed by Western blotting of cytosolic and nuclear fractions (Fig. 2D). In the cytosolic fractions the relative amount of p21^Cip1/WAF1^ was similar in control and PKCδWT cells. Furthermore, the cell cycle inhibitor remained cytosolic in PKCδWT cells also after synchronization of cells by serum removal (Fig. S2). Although an increased number of nuclei of PKCδKN INS-1E cells stained positive for p21^Cip1/WAF1^, the amount of p21^Cip1/WAF1^ protein detected on Western blots was not increased relative to the amount of nuclear proteins (data not shown). These findings do also support the hypothesis that PKCδWT cells proliferate faster due to reduced p21^Cip1/WAF1^ activity.

**Figure 3:**
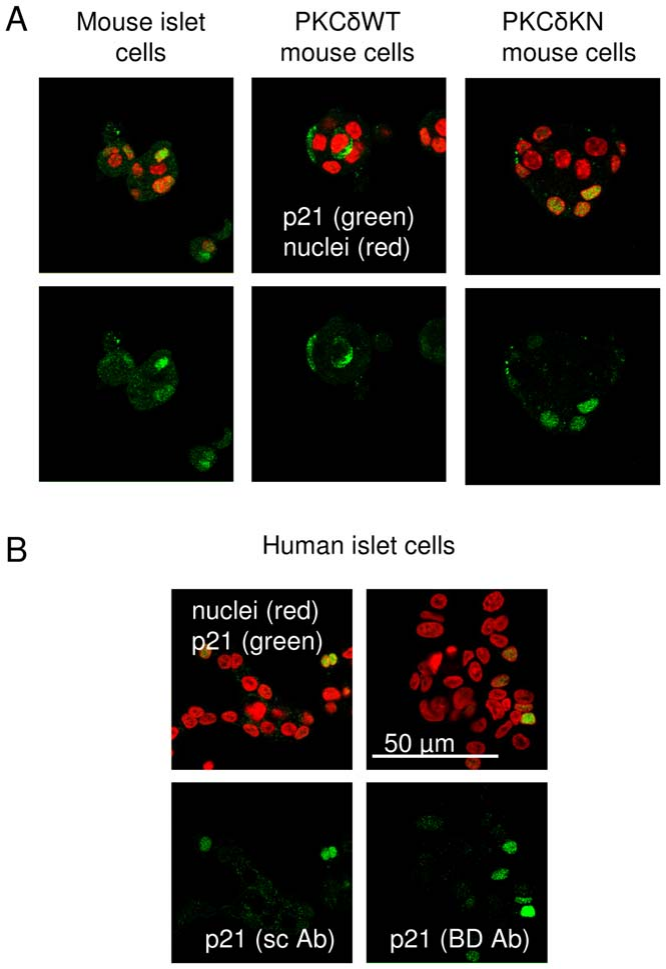
Expression of p21^Cip1/WAF1^ in mouse and human islets and PKCδ-dependent changes in cytosolic-nuclear distribution in mouse islet cells. (A) Shown are representative pictures of immunocytochemical staining for p21^Cip1/WAF1^ in islet cells of control, PKCδWT and PKCδKN transgenic mice. Nuclei are stained in red, p21^Cip/WAF1^ in green. Note the absence of nuclear staining of p21^Cip/WAF1^ in PKCδWT mouse islet cells. (B) Immunohistochemical detection of p21^Cip/WAF1^ by laser scanning microscopy in cultured human islet cells using two distinct antibodies from Cell Signalling (sc Ab) and BD Biosciences (BD Ab).

To transfer this finding to native β-cells, proliferation of islet cells was examined in pancreatic slices of WT and β-cell specific PKCδWT transgenic mice. Even after high fat feeding, Ki67 staining was not detectable neither in WT nor in PKCδWT β-cells, which suggests that proliferation remained low (data not shown). However, p21^Cip1/WAF1^ immunoreactivity was found in nuclei of cultured mouse and human islet cells (Fig. 3A and 3B). Similarly, mice islet cells with targeted expression of PKCδWT in β-cells showed reduced nuclear accumulation of p21^Cip1/WAF1^, whereas a prominent nuclear staining was evident in islet cells from control mice and PKCδKN transgenic mice (Fig. 3A). These observations suggest that PKCδ-dependent regulation of p21^Cip1/WAF1^ might also influence proliferation of native β-cells.

### PKCδ-dependent phosphorylation of p21^Cip1/WAF1^ at Ser146 regulates its subcellular distribution and function

**Figure 4:**
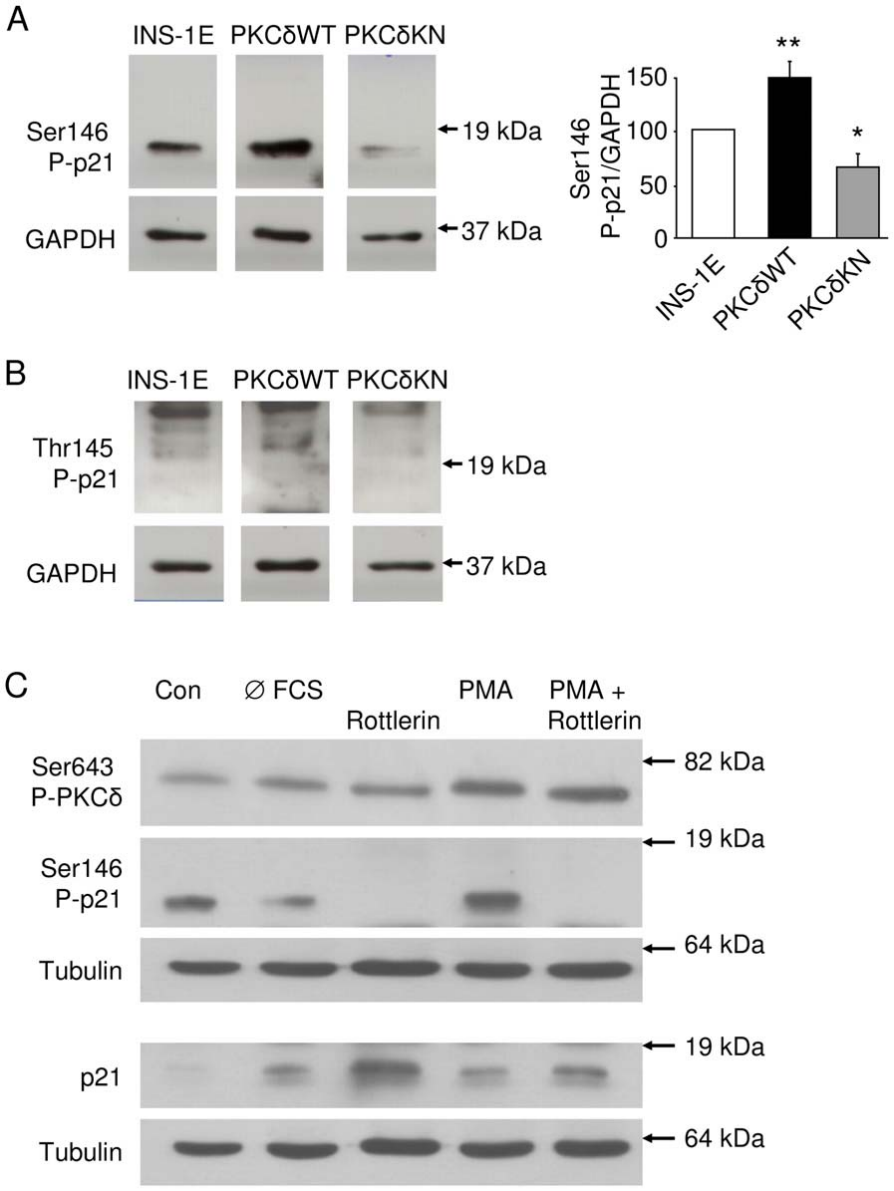
Phosphorylation of p21^Cip1/WAF1^ depends on PKCδ activity. Western blot analysis of cell lysates from control, PKCδWT and PKCδKN INS-1E cells for p21^Cip1/WAF1^ phosphorylation at (A) Ser146 and (B) Thr145. The densitometric analysis of phospho-Ser146 staining of p21^Cip1/WAF1^ to GAPDH as loading control is given as means ± SEM of n = 3 independent experiments. * (p<0.05) and ** (p<0.01) indicate significance to the respective band of INS-1E cells set to 100%. (C) Western blots for Ser643 P-PKCδ, Ser146 P-p21^Cip1/WAF1^, p21^Cip1/WAF1^ and tubulin as loading control of cell lysates from INS-1E cells after 2 d control culture (Con), after serum starvation for 16 h (Ø FCS), after 90 min treatment with the PKCδ inhibitor rottlerin (10 µM) and after stimulation with PMA (1 µM for 2 min) in the presence or absence of rottlerin (10 µM for 90 min).

**Figure 5:**
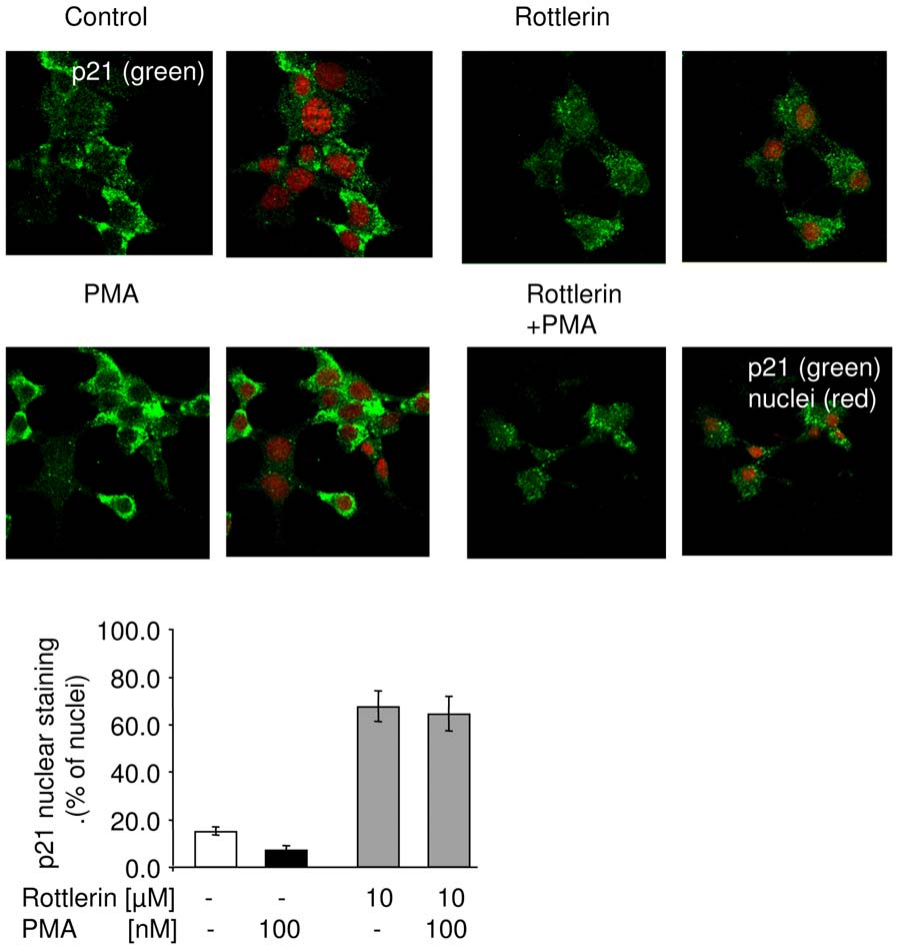
Effects of rottlerin and phorbol ester on cellular distribution of p21^Cip1/WAF1^. Representative pictures of immunocytochemical staining for p21^Cip1/WAF1^ in INS-1E cells after 16 h serum starvation (control), after 90 min treatment with the PKCδ inhibitor rottlerin (10 µM) and after stimulation with PMA (1 µM for 2 min) in the presence or absence of the PKCδ inhibitor rottlerin (10 µM) for 90 min.

To investigate the molecular mechanism of PKCδ-dependent subcellular distribution of p21^Cip1/WAF1^, the phosphorylation of p21^Cip1/WAF1^ at two regulatory sites was examined next. In comparison to control INS-1E cells phoshorylation of p21^Cip1/WAF1^ at Ser146 was significantly increased in PKCδWT cells, while it was reduced in PKCδKN cells (Fig. 4A). Phosphorylation of Thr145 was not detectable (Fig. 4B). To substantiate the effect of PKCδ on p21^Cip1/WAF1^, its phosphorylation at Ser146 was examined in cells stimulated with the phorbol myristate acetate (PMA). When cells were starved overnight, phosphorylation at Ser146 declined (Fig. 4C, first and second line). PMA stimulated p21^Cip1/WAF1^ phosphorylation in starved cells, an effect that was completely abolished by the PKCδ inhibitor rottlerin. Inhibition of phosphorylation was accompanied by an increase in p21^Cip1/WAF1^ protein (Fig. 4C). Moreover, the effects were specific for PKCδ, as inhibition of protein kinase B or ERK1/2 neither inhibited phosphorylation at Ser146 nor promoted nuclear accumulation of p21^Cip1/WAF1^ (Fig. S3). As a matter of fact nuclear staining of p21^Cip1/WAF1^ was reduced after stimulation of PKCs with PMA and was increased after treatment of INS-1E cells with rottlerin (Fig. 5). Similar but less pronounced results were obtained with PKCδWT INS-1E cells (data not shown). Although rottlerin is a sensitive inhibitor of PKCδ, it also affects other kinases such as CaM kinase III [35]. Therefore, we used the more specific siRNA-approach to reduce PKCδ expression and activity.

**Figure 6:**
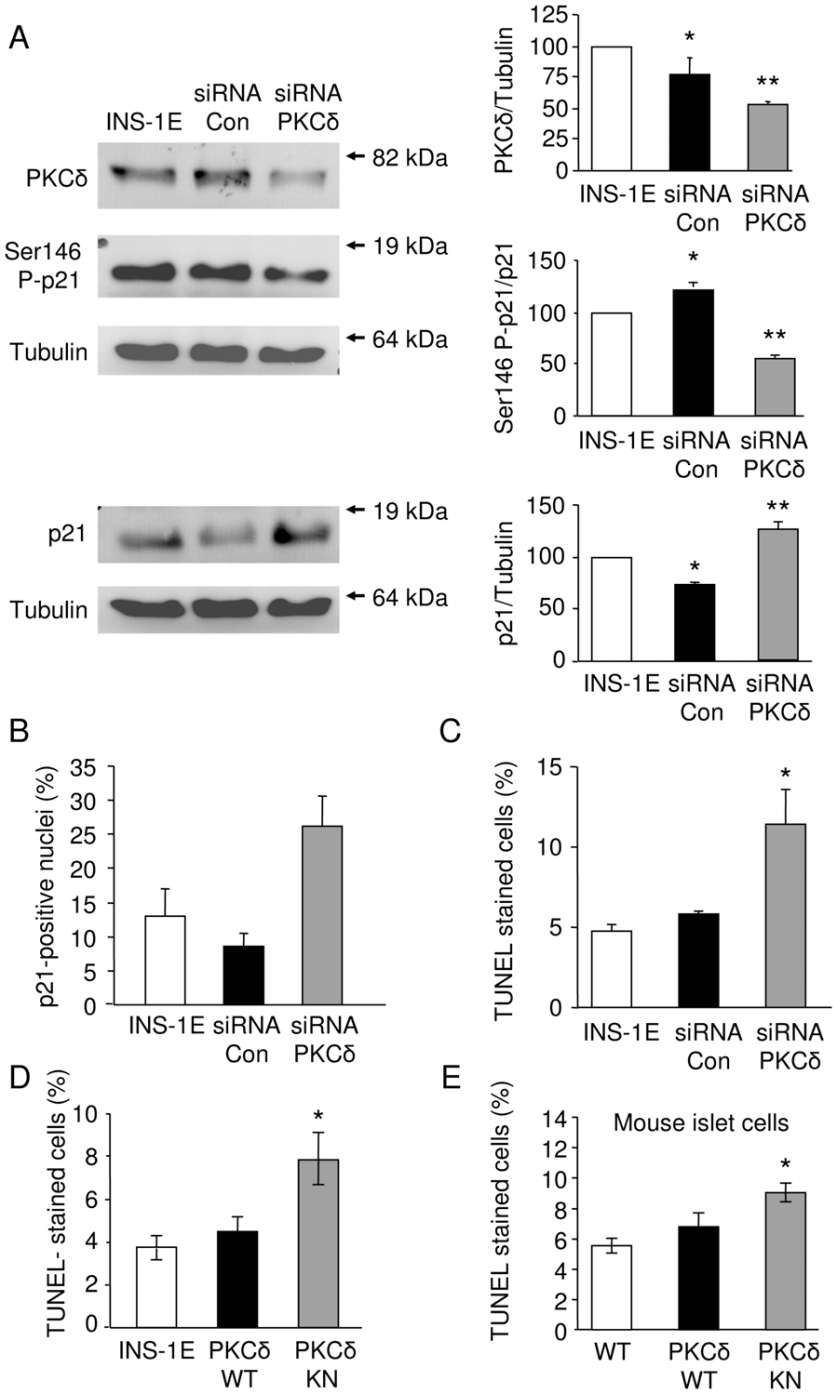
Cells treated with siRNA against PKCδ accumulate dephosphorylated p21^Cip1/WAF1^ in nuclei and display increased apoptosis. (A) Representative Western blots of PKCδ, phospho-Ser146 p21^Cip1/WAF1^ and p21^Cip1/WAF1^ of control INS-1E cells and INS-1E cells treated with control siRNA or PKCδ-specific siRNA and the respective densitometric analysis presented as means + SEM of n = 3 independent experiments. * (p<0.05) and ** (p<0.01) indicate significance to the respective band of control INS-1E cells set to 100%. (B) Percentage of p21^Cip1/WAF1^ positive nuclei analyzed by laser scanning microscopy is expressed as means + SEM of n = 3–4 independent experiments. (C–E) Percentage of TUNEL positive INS-1E cells treated with siRNA (C), control INS-1E cells, PKCδWT and PKCδKN INS-1E cells (D) and islet cells isolated from control mice, PKCδWT and PKCδKN transgenic mice (E) expressed as means + SEM of n = 3 independent experiments. * (p<0.05) indicates significance to the number of TUNEL-positive control INS-1E cells.

In cells transfected with siRNA against PKCδ a significant reduction of PKCδ expression was accompanied by a reduced phosphorylation at Ser146 and a concomitant increase in the protein amount of p21^Cip1/WAF1^ (Fig. 6A), an effect not found in cells treated with control siRNA. Consistent with the results obtained with PKCδKN cells, the knockdown of PKCδ by siRNA resulted in an increased nuclear translocation of p21^Cip1/WAF1^ (Fig. 6B).

These data strongly suggest that p21^Cip1/WAF1^ is a substrate of PKCδ in insulin-secreting cells. Phosphorylation of p21^Cip1/WAF1^ by PKCδ results in its nuclear extrusion and thereby may support proliferation.

### Functional consequences of reduced PKCδ expression in insulin-secreting cells under stress-free conditions

As PKCδ supports fatty acid induced apoptosis, the effect of changes in PKCδ expression on cell death was examined in more detail. Surprisingly, inhibition of endogenous PKCδ with siRNA or with PKCδKN mutant almost doubled the incidence of apoptotic cell death under non-stress conditions, as revealed by TUNEL staining (Fig. 6C and Fig. 6D). In accordance, isolated islet cells from transgenic mice expressing PKCδKN in β-cells displayed increased TUNEL staining when compared to control mice (Fig. 6E). In contrast, overexpression of PKCδWT in INS-1E cells and mice β-cells did not stimulate apoptosis under control culture conditions (Fig. 6D and 6E). These observations suggest that PKCδ *per se* is not pro-apoptotic but rather promotes proliferation.

Finally, the impact of changes in PKCδ expression on cell cycle was examined. When cells were stained for the G2/M marker phospho-Ser10 histone H3, the same amount of control and PKCδWT nuclei (7%), but significant more PKCδKN nuclei (17%) stained positive for phospho-Ser10 histone H3 (Fig. S4). Cell cycle analysis by flow cytometry of propidium iodide-stained nuclei revealed two distinct DNA peaks (Fig. S5A). While the major peak of control cells (58%) and significant more PKCδWT INS-1E cells (70%) showed similar DNA staining which represents G1 (2n chromosomes), a minor part of the cells resided in G2 (4n). The first DNA peak of PKCδKN cells was visible at 4n (80%) and the second peak at 8n (20%), which probably represent cells with increased DNA content at G1 and G2, respectively. That the DNA peaks correlate to 2n, 4n and 8n was confirmed by treatment of the cells with colchicine (0.5 µM for 2 d) which arrests cell cycle at G2/M transition (Fig. S5B). When DNA from freshly isolated mouse islet cells was examined, more than 95% of WT and PKCδKN cells stained for 2n and less than 2% of the cells for G2 (Fig. S6). The prominent peak (2n) of WT and PKCδKN islet cells suggests that mouse islet cells are arrested in G0/G1 and that PKCδKN expression did not affect the arrest.

This study deciphers a direct link between PKCδ and the cell *cycle* inhibitor p21^Cip1/WAF1^ which may influence β-cell proliferation. The mechanism which drives PKCδ from a proliferative into a pro-apoptotic role under stress conditions remains to be elucidated.

## Discussion

**Figure 7:**
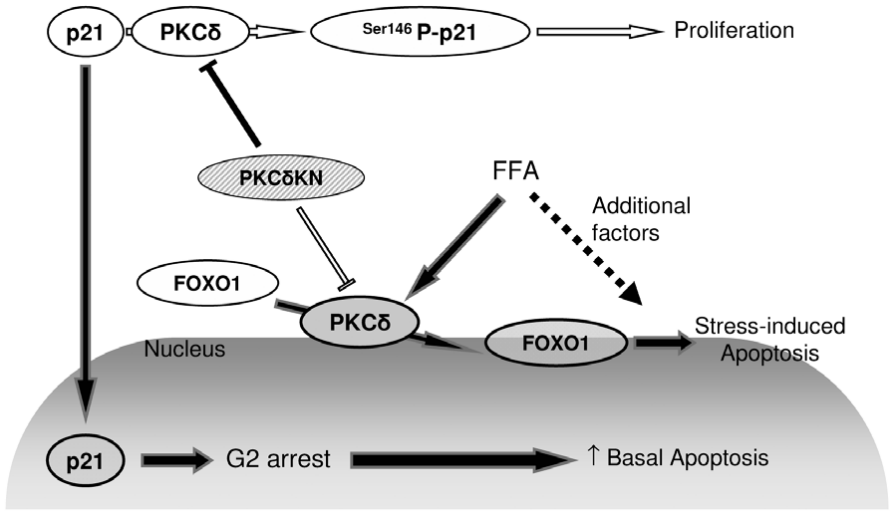
Dual effect of PKCδ in insulin-secreting cells. PKCδ supports proliferation by phosphorylation of p21^Cip/WAF1^ which results in nuclear extrusion of the cell cycle inhibitor (white symbols). In the presence of metabolic stress, e.g. prolonged exposure to high concentrations of free fatty acids (FFA) PKCδ favors apoptotic cell death through nuclear accumulation and stimulation of FOXO1 (gray symbols). PKCδKN (white and gray stripes) inhibits both the anti-apoptotic and pro-apoptotic effect of PKCδ.

The present study discloses the cell cycle inhibitor p21^Cip1/WAF1^ as a target of PKCδ in insulin-secreting cells. Phosphorylation of p21^Cip1/WAF1^ at Ser146 by PKCδ leads to its nuclear extrusion, thereby favoring cell proliferation and survival. The fact that p21^Cip1/WAF1^ is a substrate of PKCδ is consistent with a previous report [20] and supported by our observation that both RNA interference as well as a pharmacological inhibitor of PKCδ suppressed phosphorylation of p21^Cip1/WAF1^, whereas the PKC activator PMA increased p21^Cip1/WAF1^ phosphorylation. Furthermore, inhibition of PKCδ activity by expression of a kinase-inactive PKCδ mutant reduced phosphorylation and increased nuclear accumulation of p21^Cip1/WAF1^. In contrast, in PKCδWT-expressing cells p21^Cip1/WAF1^ was phosphorylated at Ser146 and largely confined to the cytoplasm. Interestingly, inhibition of PKB and ERK1/2 did not diminish phosphorylation of the cell cycle inhibitor, indicating that PKCδ is the major regulator of p21^Cip1/WAF1^ in insulin-secreting cells. Thus, our data suggest that in proliferating insulin-secreting cells PKCδ supports proliferation, at least in part, by reducing nuclear accumulation and stability of p21^Cip1/WAF1^ (Fig. 7). In pancreatic slices of PKCδWT mice, proliferation was not detectable. Similarly, in p21^Cip1/WAF1^ KO mice, proliferation of pancreatic islet cells was also not significantly increased (0.4% in WT and 0.6% in KO) [22]. These observations suggest that p21^Cip1/WAF1^ does not induce proliferation. Indeed, cell cycle inhibitors rather influence the velocity of proliferation while induction of β-cells proliferation occurs only under special conditions such as in new born and pregnant and lactating animals or after 90% pancreatectomy.

PKCδ has been found to be involved in a variety of cellular events. Although several reports indicate a pro-apoptotic role, PKCδ was also shown to exert anti-apoptotic and proliferative effects in various cell types. Such opposing effects of PKCδ may be cell type- or stimulus-specific or mediated by spatio-temporal differences of PKCδ activation. There is evidence that, similar to p21^Cip1/WAF1^, different functional effects of PKCδ are connected with the diverse compartmentalization of the enzyme. Upon stimulation with phorbol ester or fatty acids, PKCδ redistributes between a cytosolic, a membrane-bound and a cytoskeleton-associated compartment in β-cells [36]. The pro-apoptotic effect of PKCδ is linked to its nuclear accumulation [37], [38]. Furthermore, cleavage of PKCδ by caspase-3 releases a constitutively active fragment that promotes apoptosis [39]. Our data suggest that a substantial amount of PKCδWT in transgenic cells is phosphorylated at Ser643, an autophosphorylation site and, consequently, is stimulated under control culture conditions. This increased activity does not induce apoptotic cell death suggesting that additional factors generated under stress conditions are needed to turn PKCδ into a pro-apoptotic kinase (Fig. 7). Reduced PKCδ activity significantly augmented apoptosis consistently in PKCδKN INS-1E cells (up to 2-fold), in PKCδKN transgenic mouse β-cells (by 60%), as well as after down regulation of PKCδ by RNA interference in control INS-1E cells (2-fold). Although not discussed in detail, in a study by Cantley et al. using PKCδKO mice, the rate of apoptosis was 80% higher in knockout cells than in control cells [11]. The physiological impact of this finding remains unclear, especially as islet size and insulin content were not reduced in PKCδKO mice when compared to wild type mice [40]. In agreement, mice that express PKCδKN exclusively in β-cells show no reduction in islet size and insulin content [14], [41].

In addition to cell cycle regulation, emerging evidence suggests that p21^Cip1/WAF1^ exerts other functions in diverse cellular processes, including cell differentiation and survival. The impaired replication and increased apoptosis of β-cells of p21^Cip1/WAF1^ transgenic mice mirror our observations obtained in insulin secreting cells with reduced PKCδ activity and may thus result from prolonged nuclear accumulation of p21^Cip1/WAF1^ [29]. Interestingly, similar to PKCδKO mice, these mice show improved recovery from streptozotocin-induced hyperglycemia, which has been attributed to an increased regeneration of insulin-producing cells [11], [29]. In line, our previous study disclosed protection of mice with β-cell specific expression of PKCδKN against HFD-induced hyperglycemia. Apparent contradictory to this assumption are two studies which link stress-induced expression of p21^Cip1/WAF1^ to reduced insulin mRNA and β-cell failure. In one study oxidative stress-induced expression or exogenous overexpression of p21^Cip1/WAF1^ in rat islets suppressed insulin biogenesis [42]. In the second study using a mouse model with deficient DNA repair reduced β-cell proliferation and the onset of diabetes was accompanied by increased expression of p21^Cip1/WAF1^ [43]. One difference which might explain these opposing results between the two latter studies and our cell models is the persistent expression of p21^Cip1/WAF1^, while in our cell systems endogenous p21^Cip1/WAF1^ expression is transient.

Phosphorylation events presumably not only regulate the compartmentalization but also the protein stability of p21^Cip1/WAF^, although controversial data have been reported on this issue. It was previously shown that PKB-dependent phosphorylation of p21^Cip1/WAF1^ at both Thr145 and Ser146 increases the stability of the CDK inhibitor and enhances its anti-apoptotic activity [31], [44], [45]. However, in our experiments, inhibition of PKB by Akti-1/2 affected neither phosphorylation nor nuclear accumulation of p21^Cip1/WAF1^, suggesting a minor role of PKB in our cell system. Likewise, inhibition of ERK1/2 with PD98059 had no impact on the subcellular distribution and phosphorylation of p21^Cip1/WAF1^. Although we did not study the effect of PKCδ on p21^Cip1/WAF1^ stability in detail, PKCδWT cells revealed a significantly lower amount of p21^Cip1/WAF1^ than control or PKCδKN cells, suggesting that Ser146 phosphorylation affects p21^Cip1/WAF1^ stability. These data are consistent with reports demonstrating that phosphorylation of p21^Cip1/WAF1^ at Ser146 by PKCδ leads to destabilization of the CDK inhibitor [46].

Thus, our data indicate that p21^Cip1/WAF1^ exerts a dual effect depending on its subcellular distribution (Fig. 7). When trapped in the cytosol due to phosphorylation, p21^Cip1/WAF1^ might favor proliferation, a notion supported by the increased Ki67 staining in PKCδWT cells. This cytosolic localization of p21^Cip1/WAF1^ is known to exert anti-apoptotic effects by CDK-dependent or independent mechanisms [47], [48]. One mechanism of anti-apoptotic action of p21^Cip1/WAF1^ involves its direct binding to and inhibition of the pro-apoptotic kinases ASK1 or JNK [31], [49], [50].

In contrast, nuclear p21^Cip1/WAF1^ inhibits cell cycle progression and might eventually lead to apoptosis. In addition to the binding to cyclin-CDK complexes, p21^Cip1/WAF1^ interacts directly with the proliferating cell nuclear antigen (PCNA), and thereby inhibits PCNA-dependent DNA replication [51]. Improper and prolonged nuclear accumulation of p21^Cip1/WAF1^ may explain the observations that mice overexpressing p21^Cip1/WAF1^ specifically in β-cells develop age-related hyperglycemia under normal feeding [29].

Whether PKCδ-dependent regulation of p21^Cip1/WAF1^ affects β-cell function in humans needs further experimental evidence. The expression of p21^Cip1/WAF1^ in human islets and the fact that PKCδ reduces nuclear accumulation of p21^Cip1/WAF1^ in primary mouse islet cells supports the view that the cell cycle inhibitor could play a regulatory role also in adult human β-cells under special proliferative conditions [23].

In conclusion, our study demonstrates that PKCδ induces posttranslational modifications of p21^Cip1/WAF1^ which, in turn, determine its subcellular distribution and function in INS-1E cells. This study reveals that PKCδ is not per se a pro-apoptotic kinase and underlines the importance of understanding molecular mechanisms for the evaluation of therapeutic targets in the treatment of diabetes mellitus.

## Materials and Methods

### Ethics Statement

The use for scientific purposes of isolated human islets was approved by the local ethics committee (University of Tuebingen, Medical Faculty No. 533/2010BO2). All animal experiments were done in accordance with the accepted standard of human care of animals and approved by the local Animal Care and Use Committee (Notification from 12.01.10).

### Cell preparations, culture and transfection

INS-1E cells, kindly provided by C. B. Wollheim (University of Geneva, Switzerland), were cultured in RPMI 1640 (GIBCO) containing 11 mM glucose, 2 mM L-glutamine, 10 mM HEPES, 1 mM sodium pyruvate, 0.05 mM β-mercaptoethanol and 10% FCS as described [52]. INS-1E cells were stably transduced with retroviruses encoding wild type PKCδ (PKCδWT) or the K376R-mutation in the ATP-binding domain (PKCδKN) driven by the rat insulin 1 gene promoter. Cell clones expressing the transgene were selected by geneticin (G418) and subcloned as single cell clones. INS-1E cells were transfected with siRNA against PKCδ (20 nM/5×10^5^ cells; On-Target plus siRNA, # J-080142-05, Dharmacon, Chicago) using a siRNA transfection reagent (DharmaFECT 3, Dharmacon). As control, siRNA against luciferase (20 nM/5×10^5^ cells) was transfected in parallel. Cells were used 2 d after transfection. p21^Cip1/WAF1^-proficient and deficient mouse embryonic fibroblasts (MEFs) were cultured in DMEM supplemented with 10% FCS and antibiotics.

Human islet preparations were obtained from the ECIT (European Consortium of Islet Transplantation) Center in Geneva (Switzerland). Human islets, purified by hand picking, were digested to single cells with trypsin (40 units/ml trypsin-EDTA in PBS) for 4–6 min at 37°C. Isolated islet cells were then cultured in CMRL 1066 medium (GIBCO, Invitrogen GmbH, Karlsruhe, Germany) containing 5.5 mM glucose, 10% fetal calf serum (FCS, Biochrom, Berlin, Germany), 2 mM L-glutamine and 10 mM HEPES. After 2 d culture on collagen (2 µg/ml human collagen type 1) coated glass cover slips cells were used for immunocytochemical staining. Transgenic mice were generated and isolated islet cell culture was prepared as described previously [14], [53].

### Cell cycle analysis by Nicoletti

After 2 d culture, cells were detached by trypsin and resuspended in Nicoletti buffer containing 0.1% sodium citrate, pH 7.4, 0.1% Triton X-100 and 50 µg/ml propidium iodide. DNA staining was analyzed by flow cytometry using the FL2-H channel.

### Immunocytochemistry and TUNEL staining

Isolated mouse islet cells, INS-1E cells and MEFs were cultured for 2 d on L-poly-ornithine (0.001%) coated glass cover slips. Cells were fixed with 4% paraformaldehyde in phosphate-buffered saline (PBS), permeabilized with 0.2% Triton X-100 and preincubated in 10% FCS-PBS for 45 min. Primary antibodies against phospho-Ser10 histone H3 (1∶150, from Millipore, Billerica, MA), p21^Cip1/WAF1^ (rabbit polyclonal antibody, 1∶200, from Santa Cruz Biotechnology, Santa Cruz, CA or mouse monoclonal antibody, 1∶150, from Becton Dickinson, Heidelberg, Germany), Ki67 (1∶50, DakoCytomation, Hamburg, Germany) were applied overnight in 10% FCS-PBS. After 30 min washing with FCS-PBS the cells were incubated for 1 h with the secondary antibody in 10% FBS-PBS (Alexa-Fluor488 coupled anti-rabbit or anti-mouse IgG, 1∶400, Invitrogen GmbH, Darmstadt, Germany). Thereafter, nuclei were stained with 1 µM TOPRO-3 (Invitrogen) in PBS for 1 h. The fluorescence was examined with a confocal laser scanning microscope (Leica, Wetzlar, Germany). For TUNEL staining cells were prepared according to the protocol provided by the commercial kit (Roche Diagnostics, Mannheim, Germany).

### Western blotting

Islets and INS-1E cells were lysed in buffer containing 125 mM NaCl, 1% Triton X-100, 0.1% SDS, 10 mM EDTA, 25 mM HEPES pH 7.3, 10 mM NaPP, 10 mM NaF, 1 mM Na-vanadate, 10 µg/ml pepstatin A, 10 µg/ml aprotinin and 0.1 mM PMSF. Protein concentrations of cell lysates were determined using the Bradford dye-binding procedure from Biorad Laboratories (Munich, Germany). Cytosolic and nuclear fractions of INS-1E cells were prepared using a commercial kit (Pierce Biotechnology, Rockford IL). Cell homogenates or cytosolic and nuclear fractions were subjected to SDS-PAGE (8–12%) and blotted onto nitrocellulose membranes (Schleicher & Schuell, Dassel, Germany). Membranes were incubated overnight with primary antibodies (diluted 1∶1000 in TBS-Tween containing 5% BSA or 5% milk powder) followed by incubation with horseradish peroxidase-coupled anti-rabbit IgG (1∶2000 in TBS-Tween, 5% milk powder). Antibodies against PKCδ, phospho-Thr505 and phospho-Ser643 PKCδ, p27 and tubulin were from Cell Signaling Technology (Danvers, MA), antibodies against GAPDH, histone H1, and polyclonal antibodies against p21^Cip1/WAF1^ and phospho-p21^Cip1/WAF1^ (phospho-Ser146, phospho-Thr145) were from Santa Cruz Biotechnology.

### Statistics

Data are expressed as means ± SEM, p<0.05 (unpaired Student's t-test or 2-way ANOVA followed by Bonferroni post test where applicable) was considered significant.

## Supporting Information

Figure S1 **Specificity controls of the p21^Cip1/WAF1^ antibodies.** (A) Shown is a Western blot of homogenates from p21^Cip1/WAF1^ proficient or deficient MEFs that were either left untreated or incubated with the DNA-damaging agent bleomycin (BLM, 10 µM for 20 h). On Western blots p21^Cip1/WAF1^ migrates at an apparent molecular weight of 17–18 kD. Tubulin was used as loading control. (B) Immunocytochemical staining of p21^Cip1/WAF1^ in p21^Cip1/WAF1^-proficient and deficient MEFs cultured under control conditions (con) or in the presence of bleomycin (BLM) using antibodies from Santa Cruz (left pictures) or Becton Dickinson (BD, right pictures).
(TIF)

Figure S2 **Cell cycle dependent expression of p21^Cip1/WAF1^.** Shown are representative pictures of immunocytochemical staining for p21^Cip1/WAF1^ (A) 16 h after serum deprivation and (B) 32 h after re-addition of 10% serum in control, PKCδWT and PKCδKN INS-1E cells. Nuclei are stained in red, p21^Cip/WAF1^ in green. Note the absence of nuclear staining of p21^Cip/WAF1^ in PKCδWT INS-1E cells 32 h after re-addition of 10% serum.
(TIF)

Figure S3 **Phosphorylation and nuclear extrusion of p21^Cip1/WAF1^ is not mediated by PKB/Akt or ERK1/2.** (A) Western blot analysis representative for 3 independent experiments with PKCδWT cell homogenates for the status of Ser146 p21^Cip1/WAF1^ phosphorylation. Cells were cultured for the indicated time in the presence of the protein kinase B inhibitor Akti-1/2 (Akti, 5 µM) or PD98059 (PD, 10 µM), a specific inhibitor of the ERK upstream MEK kinases. (B) Immunocytochemical staining for p21^Cip1/WAF1^ (green) in PKCδWT cells that were either left untreated or incubated for 24 h in the presence of Akti-1/2 (5 µM) or PD98059 (10 µM). Nuclei are stained in red. Both inhibitors (Akti and PD98059) were effective even after prolonged cell culture. Thus, IGF-1-induced PKB phosphorylation was inhibited in the cells treated with Akti. Phorbol ester-induced phosphorylation of ERK and c-fos induction were inhibited in the cells treated with PD98059 (data not shown).
(TIF)

Figure S4 **Changes in cell cycle progression of INS-1E cell expressing PKCδKN.** Representative pictures of immunocytochemical staining for phospho-Ser10 histone H3. Nuclei are stained in red, phospho-Ser10 histone H3 in green. The percentage of positive cells is given as means ± SEM from 3–4 independent experiments. * (p<0.05) represents significance to control INS-1E cells.
(TIF)

Figure S5 **Cell cycle analysis of INS-1E cells.** Representative FACS measurements of propidium iodide-stained nuclear DNA from control INS-1E, PKCδWT and PKCδKN cells (A) after standard culture and (B) after treatment with colchicine (0.5 µM for 2 d) Results show means + SEM from n = 3–4 independent experiments. * (p<0.05) and ** (p<0.01) represent significance to the respective cell cycle phase of control INS-1E cells; ## (p<0.01) represents significance to the respective condition without colchicine treatment.
(TIF)

Figure S6 **Cell cycle analysis of isolated mouse islet cells.** Representative FACS measurements of propidium iodide-stained nuclear DNA from islet cells isolated of (A) wild type mice and (B) PKCδKN transgenic mice and means + SEM from n = 3 independent experiments.
(TIF)
