# Supplementary figures and images for: Protein Kinase C Delta (PKCδ) Affects Proliferation of Insulin-Secreting Cells by Promoting Nuclear Extrusion of the Cell Cycle Inhibitor p21Cip1/WAF1
Source: PLoS One. 2011 Dec 27;6(12):e28828. doi: 10.1371/journal.pone.0028828 (PMC3246440; doi:10.1371/journal.pone.0028828)

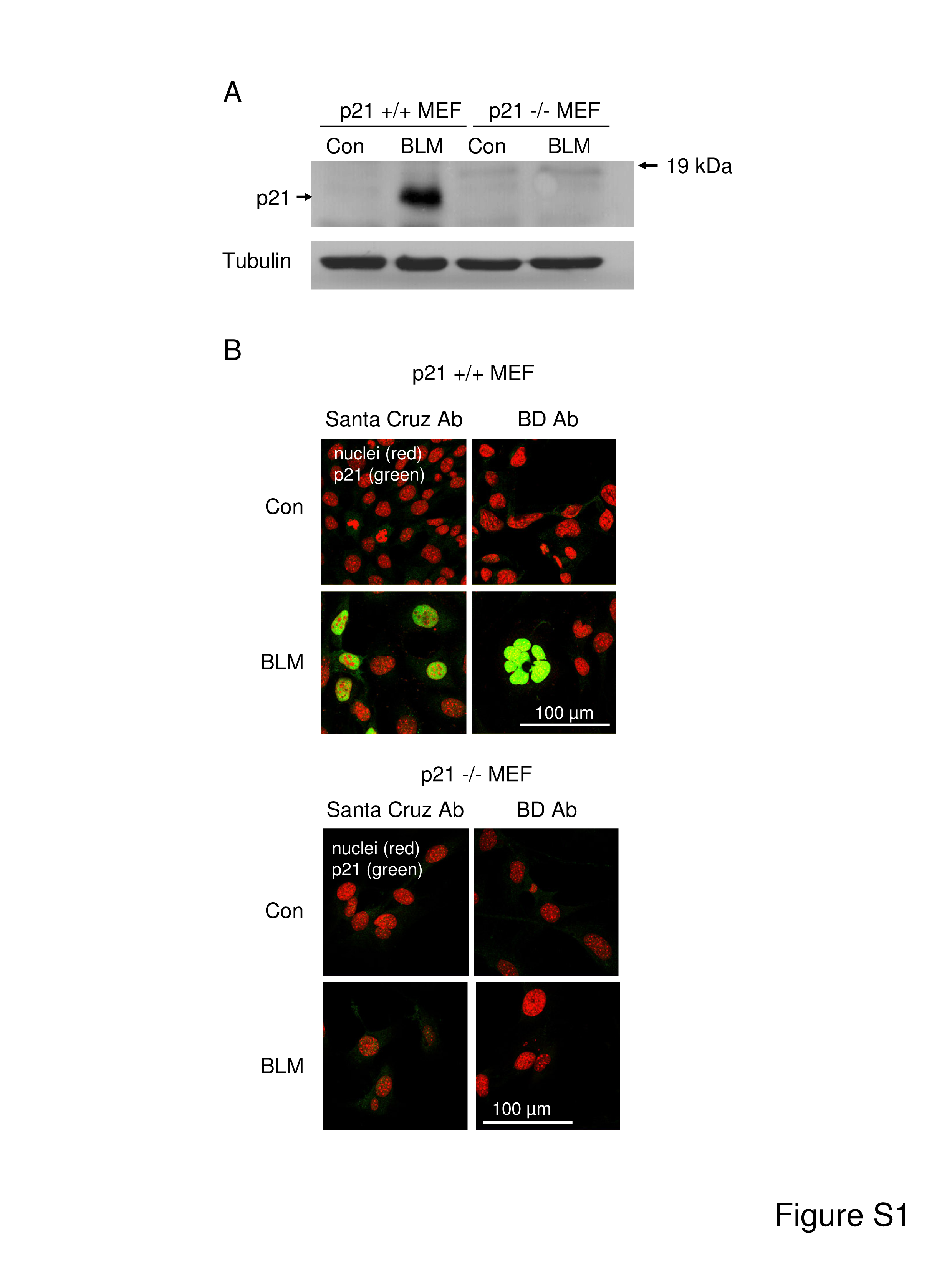

Supplement: Figure S1 — Specificity controls of the p21Cip1/WAF1 antibodies. (A) Shown is a Western blot of homogenates from p21Cip1/WAF1 proficient or deficient MEFs that were either left untreated or incubated with the DNA-damaging agent bleomycin (BLM, 10 µM for 20 h). On Western blots p21Cip1/WAF1 migrates at an apparent molecular weight of 17–18 kD. Tubulin was used as loading control. (B) Immunocytochemical staining of p21Cip1/WAF1 in p21Cip1/WAF1-proficient and deficient MEFs cultured under control conditions (con) or in the presence of bleomycin (BLM) using antibodies from Santa Cruz (left pictures) or Becton Dickinson (BD, right pictures). (TIF) [file pone.0028828.s001.tif]

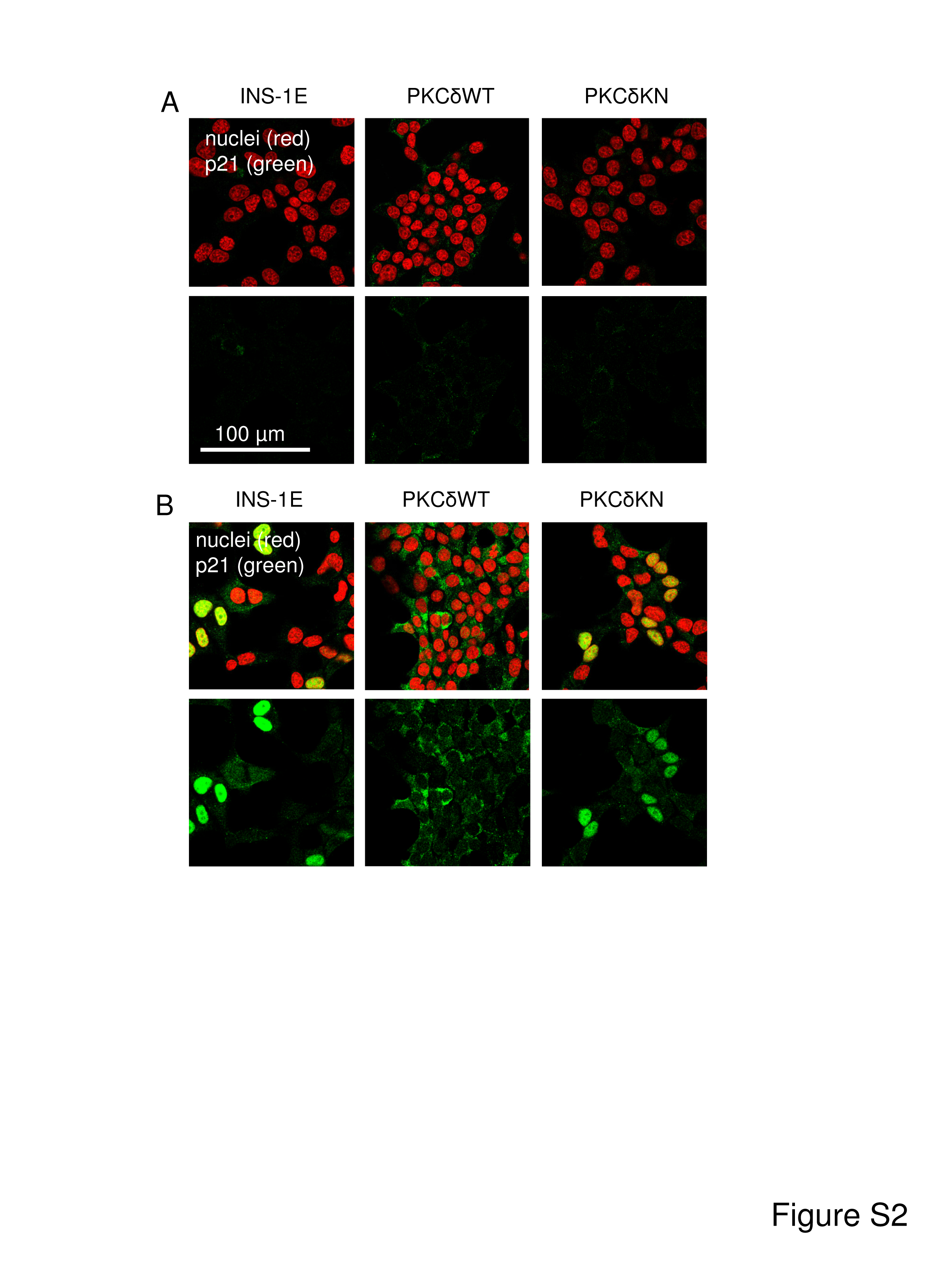

Supplement: Figure S2 — Cell cycle dependent expression of p21Cip1/WAF1. Shown are representative pictures of immunocytochemical staining for p21Cip1/WAF1 (A) 16 h after serum deprivation and (B) 32 h after re-addition of 10% serum in control, PKCδWT and PKCδKN INS-1E cells. Nuclei are stained in red, p21Cip/WAF1 in green. Note the absence of nuclear staining of p21Cip/WAF1 in PKCδWT INS-1E cells 32 h after re-addition of 10% serum. (TIF) [file pone.0028828.s002.tif]

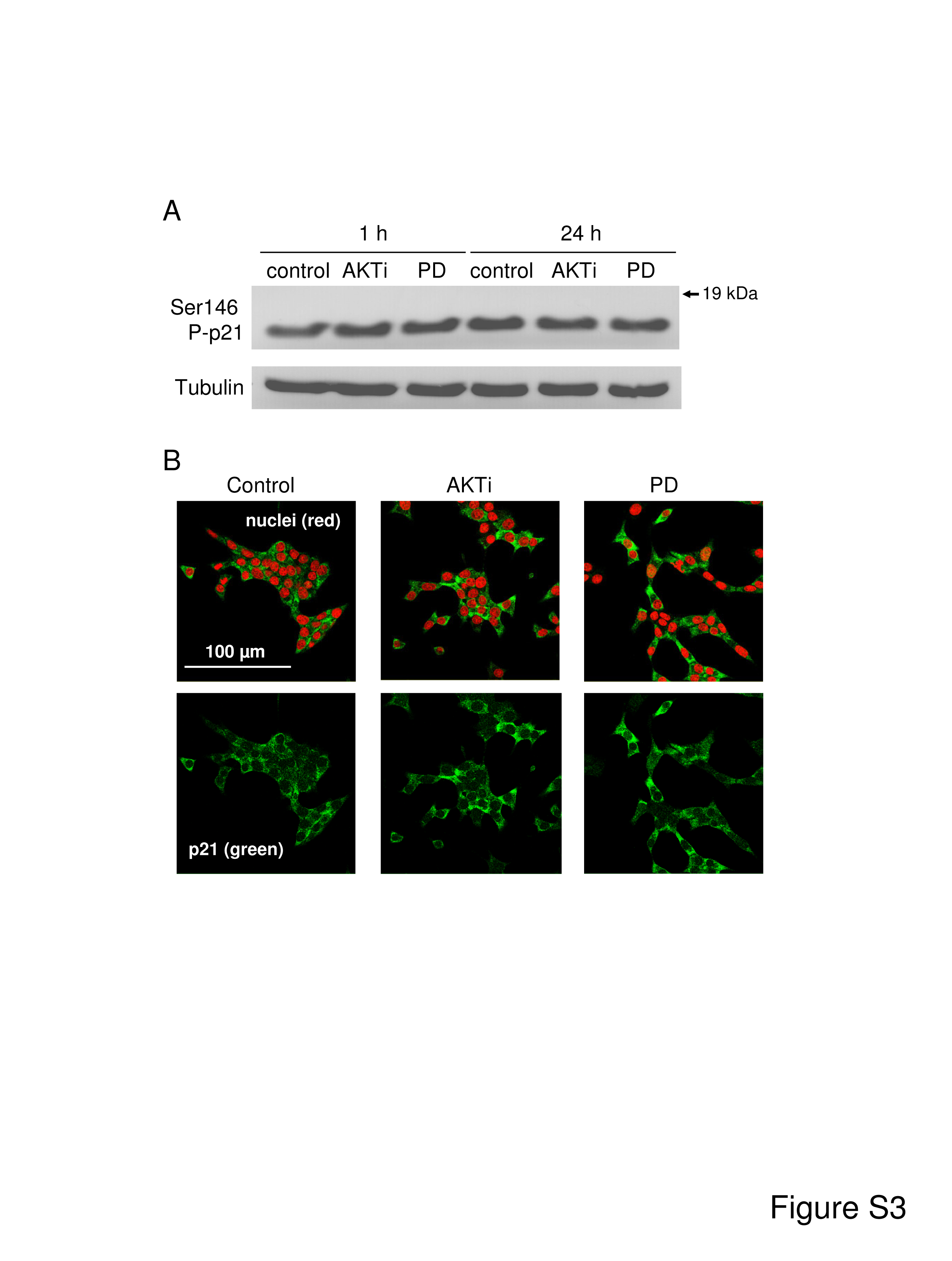

Supplement: Figure S3 — Phosphorylation and nuclear extrusion of p21Cip1/WAF1 is not mediated by PKB/Akt or ERK1/2. (A) Western blot analysis representative for 3 independent experiments with PKCδWT cell homogenates for the status of Ser146 p21Cip1/WAF1 phosphorylation. Cells were cultured for the indicated time in the presence of the protein kinase B inhibitor Akti-1/2 (Akti, 5 µM) or PD98059 (PD, 10 µM), a specific inhibitor of the ERK upstream MEK kinases. (B) Immunocytochemical staining for p21Cip1/WAF1 (green) in PKCδWT cells that were either left untreated or incubated for 24 h in the presence of Akti-1/2 (5 µM) or PD98059 (10 µM). Nuclei are stained in red. Both inhibitors (Akti and PD98059) were effective even after prolonged cell culture. Thus, IGF-1-induced PKB phosphorylation was inhibited in the cells treated with Akti. Phorbol ester-induced phosphorylation of ERK and c-fos induction were inhibited in the cells treated with PD98059 (data not shown). (TIF) [file pone.0028828.s003.tif]

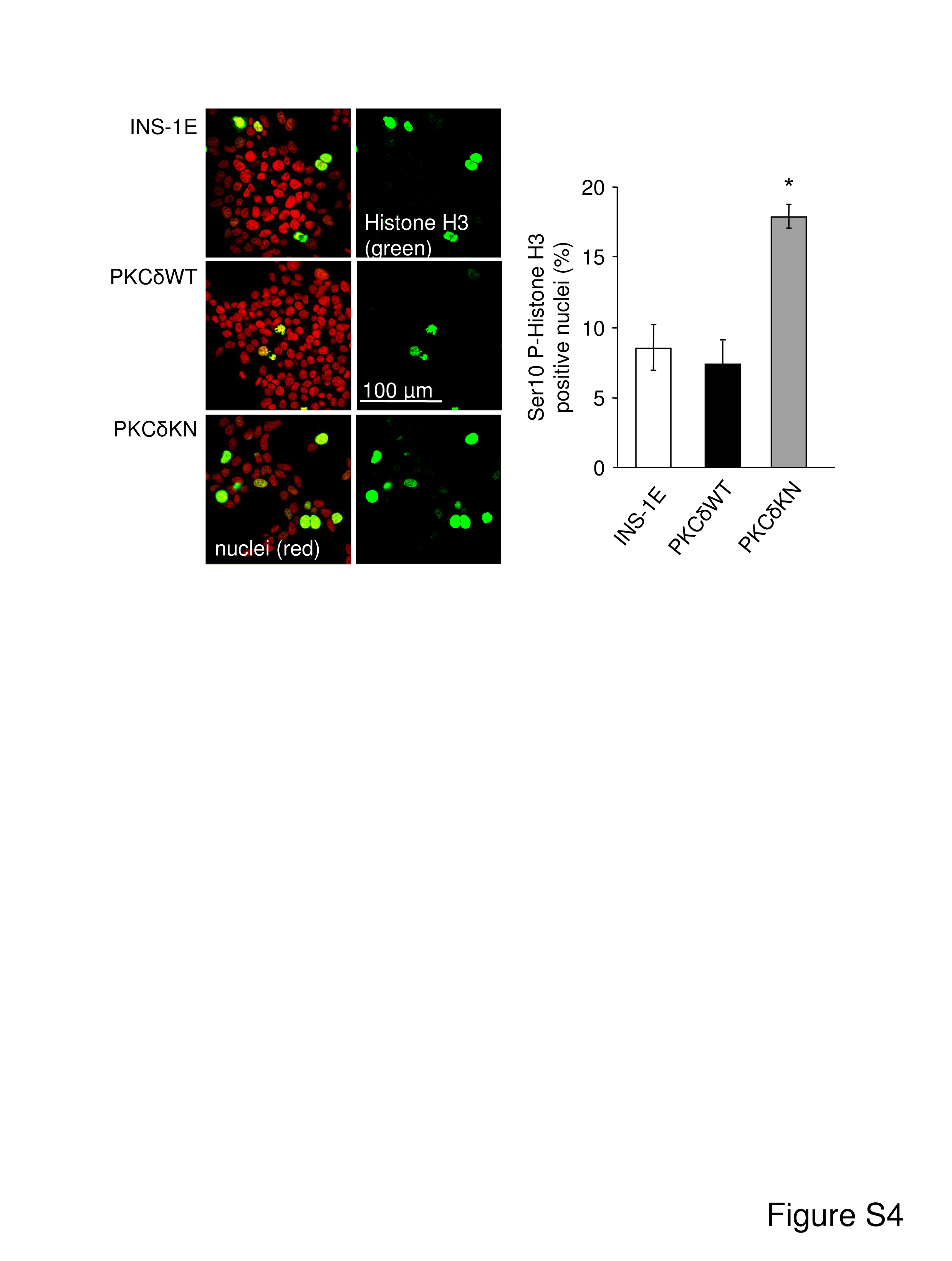

Supplement: Figure S4 — Changes in cell cycle progression of INS-1E cell expressing PKCδKN. Representative pictures of immunocytochemical staining for phospho-Ser10 histone H3. Nuclei are stained in red, phospho-Ser10 histone H3 in green. The percentage of positive cells is given as means ± SEM from 3–4 independent experiments. * (p<0.05) represents significance to control INS-1E cells. (TIF) [file pone.0028828.s004.tif]

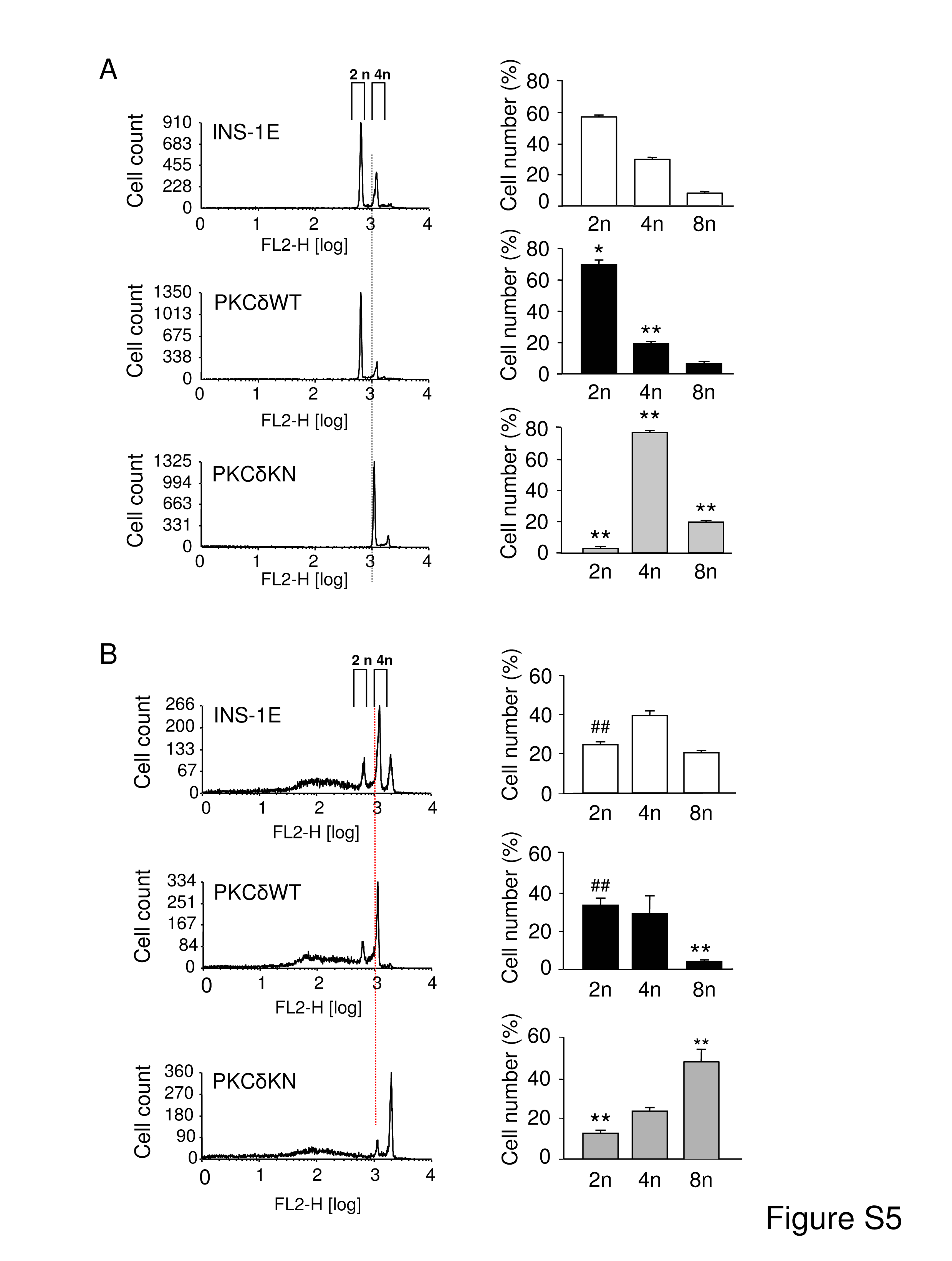

Supplement: Figure S5 — Cell cycle analysis of INS-1E cells. Representative FACS measurements of propidium iodide-stained nuclear DNA from control INS-1E, PKCδWT and PKCδKN cells (A) after standard culture and (B) after treatment with colchicine (0.5 µM for 2 d) Results show means + SEM from n = 3–4 independent experiments. * (p<0.05) and ** (p<0.01) represent significance to the respective cell cycle phase of control INS-1E cells; ## (p<0.01) represents significance to the respective condition without colchicine treatment. (TIF) [file pone.0028828.s005.tif]

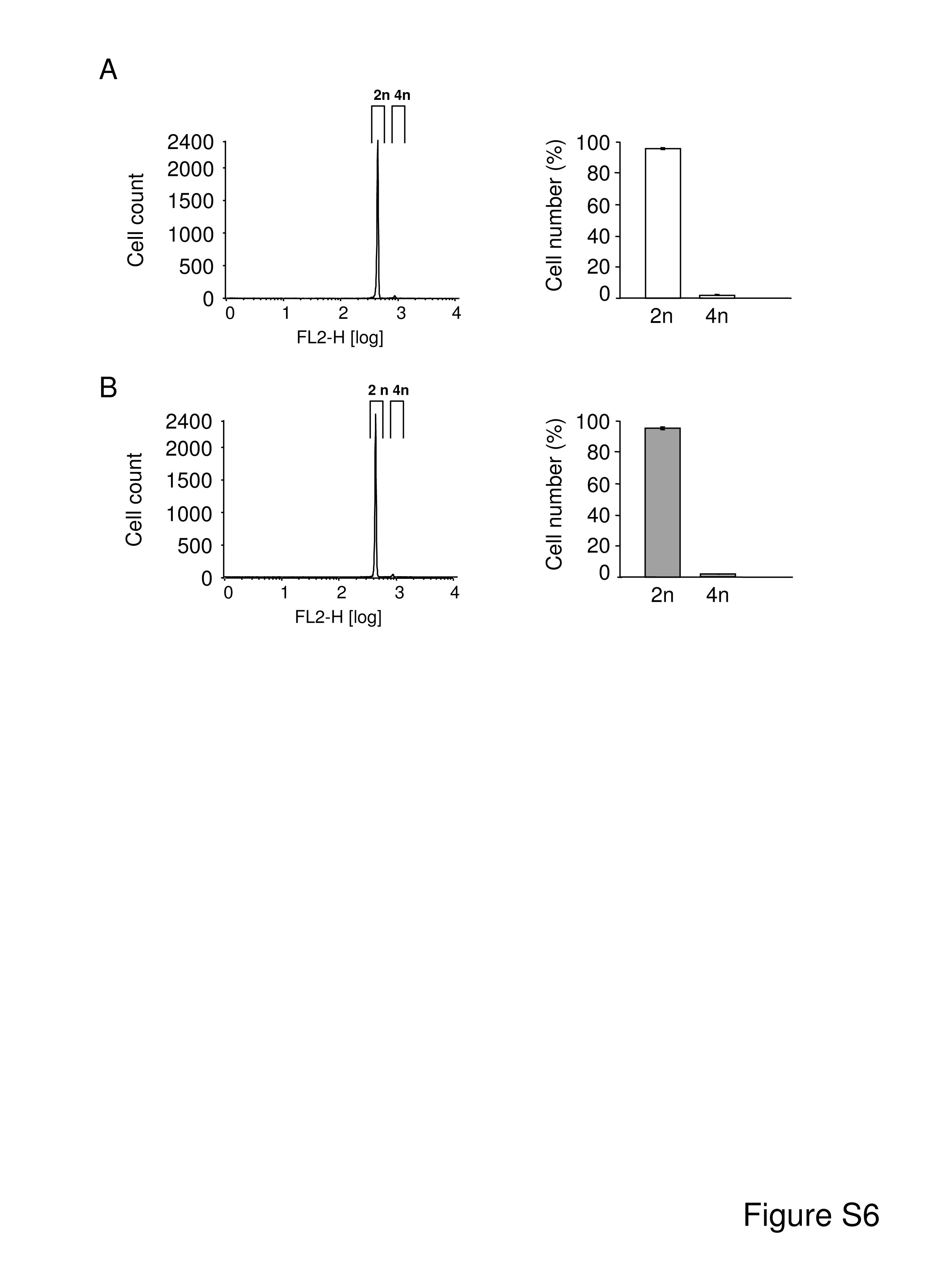

Supplement: Figure S6 — Cell cycle analysis of isolated mouse islet cells. Representative FACS measurements of propidium iodide-stained nuclear DNA from islet cells isolated of (A) wild type mice and (B) PKCδKN transgenic mice and means + SEM from n = 3 independent experiments. (TIF) [file pone.0028828.s006.tif]
